# Older adults who persistently present to the emergency department with severe, non-severe, and indeterminate episode patterns

**DOI:** 10.1186/1471-2318-11-65

**Published:** 2011-10-21

**Authors:** Brian Kaskie, Maksym Obrizan, Michael P Jones, Suzanne Bentler, Paula Weigel, Jason Hockenberry, Robert B Wallace, Robert L Ohsfeldt, Gary E Rosenthal, Fredric D Wolinsky

**Affiliations:** 1 Department of Health Management and Policy, College of Public Health, the University of Iowa, Iowa City, Iowa, USA; 2 Kyiv School of Economics, and Kyiv Economics Institute, Kyiv, Ukraine; 3 Department of Biostatistics, College of Public Health, the University of Iowa, Iowa City, Iowa; and Comprehensive Access and Delivery Evaluation and Research (CADRE) Center, Iowa City Veterans Administration Medical Center, Iowa City, USA; 4 Comprehensive Access and Delivery Evaluation and Research (CADRE) Center, Department of Health Management and Policy, College of Public Health, the University of Iowa, Iowa City, Iowa and National Bureau of Economic Research, USA; 5 Department of Health Management and Policy, Rollins School of Public Health, Emory University, Atlanta, Georgia, USA; 6 Department of Epidemiology, College of Public Health, the University of Iowa, Iowa City, Iowa, USA; 7 Department of Health Management and Policy, School of Rural Public Health, Texas A&M University Health Science Center, College Station, Texas, USA; 8 Department of Internal Medicine, Carver College of Medicine, the University of Iowa, Iowa City, Iowa, USA; 9 Department of Health Management and Policy, College of Public Health, the University of Iowa, Iowa City, Iowa; Department of Internal Medicine, Carver College of Medicine, the University of Iowa, Iowa City, Iowa; Department of Adult Nursing, College of Nursing, the University of Iowa, Iowa City, Iowa, USA

## Abstract

**Background:**

It is well known that older adults figure prominently in the use of emergency departments (ED) across the United States. Previous research has differentiated ED visits by levels of clinical severity and found health status and other individual characteristics distinguished severe from non-severe visits. In this research, we classified older adults into population groups that persistently present with severe, non-severe, or indeterminate patterns of ED episodes. We then contrasted the three groups using a comprehensive set of covariates.

**Methods:**

Using a unique dataset linking individual characteristics with Medicare claims for calendar years 1991-2007, we identified patterns of ED use among the large, nationally representative AHEAD sample consisting of 5,510 older adults. We then classified one group of older adults who persistently presented to the ED with clinically severe episodes and another group who persistently presented to the ED with non-severe episodes. These two groups were contrasted using logistic regression, and then contrasted against a third group with a persistent pattern of ED episodes with indeterminate levels of severity using multinomial logistic regression. Variable selection was based on Andersen's behavioral model of health services use and featured clinical status, demographic and socioeconomic characteristics, health behaviors, health service use patterns, local health care supply, and other contextual effects.

**Results:**

We identified 948 individuals (17.2% of the entire sample) who presented a pattern in which their ED episodes were typically defined as severe and 1,076 individuals (19.5%) who typically presented with non-severe episodes. Individuals who persistently presented to the ED with severe episodes were more likely to be older (AOR 1.52), men (AOR 1.28), current smokers (AOR 1.60), experience diabetes (AOR (AOR 1.80), heart disease (AOR 1.70), hypertension (AOR 1.32) and have a greater amount of morbidity (AOR 1.48) than those who persistently presented to the ED with non-severe episodes. When contrasted with 1,177 individuals with a persistent pattern of indeterminate severity ED use, persons with severe patterns were older (AOR 1.36), more likely to be obese (AOR 1.36), and experience heart disease (AOR 1.49) and hypertension (AOR 1.36) while persons with non-severe patterns were less likely to smoke (AOR 0.63) and have diabetes (AOR 0.67) or lung disease (AOR 0.58).

**Conclusions:**

We distinguished three large, readily identifiable groups of older adults which figure prominently in the use of EDs across the United States. Our results suggest that one group affects the general capacity of the ED to provide care as they persistently present with severe episodes requiring urgent staff attention and greater resource allocation. Another group persistently presents with non-severe episodes and creates a considerable share of the excess demand for ED care. Future research should determine how chronic disease management programs and varied co-payment obligations might impact the use of the ED by these two large and distinct groups of older adults with consistent ED use patterns.

## Background

It is well known that older adults figure prominently in the use of emergency departments (ED) across the United States [1,2]. Medicare beneficiaries over the age of 65 have the highest ED visit rates among all age groups, and their use of the ED increases by as much as 30% with advancing age [1,3]. Compared to younger adults, persons over 65 are more likely to present with complex and time-consuming health problems. They remain in the ED longer, require more intensive diagnostic work-ups, consume more treatment resources, and are more likely to be admitted to the hospital [1,4,5]. With the growing number of Americans who will reach and surpass their 65^th ^birthdays in the next decade, the demands older adults make upon the ED will become even more apparent.

Much of the previous research has examined reasons why older adults present to the ED with varying levels of severity-classifying visits as clinically severe and requiring urgent attention, being less severe, or not severe at all (and potentially unnecessary), and then associating these visits with demographic characteristics, clinical status, health behaviors, and other variables [5-12]. For example, in our previous study, we observed 18,695 ED episodes among a large sample of older Medicare beneficiaries and categorized 29.3% of the episodes as clinically severe, 33.6% as not severe, and 37.2% as indeterminate severity because they fell somewhere in between [3]. Individuals with severe ED visits typically presented with cardiac, respiratory, and other clinically severe conditions that required urgent attention. In contrast, older adults who present with non-severe conditions have been associated with contributing to the excess demand for ED care, a prominent public health problem as defined by the Institute of Medicine in the landmark Report on the Future of Emergency Care [13].

However, this previous work has relied on a limited set of covariates and supported few viable solutions other than to suggest that individuals who present to the ED should be triaged and those with non-severe conditions should be diverted - a suggestion that has been difficult to translate into clinical practice [14-16]. More recent efforts have developed predictive models in which age and disease history were identified as risk factors for increased ED use, and suggested clinical and administrative claims data could be useful in managing ED use among older individuals [17,18].

In this research, our objective was to build on these previous efforts and examine ED use from a population-based perspective. We used a unique dataset including a comprehensive set of individual characteristics linked with Medicare claims, and observed a large nationally representative sample of older adults over a 17-year period. We created three groups based on their individual patterns of ED use. The first group consisted of older adults with a persistent pattern of presenting to the ED with severe clinical conditions that required urgent care. The second group consisted of older adults who persistently presented to the ED with non-severe episodes. A third group consisted of those who had a persistent pattern of indeterminate severity episodes. We then differentiated the groups using an expanded version of Andersen's behavioral model of health services use [19] featuring covariates including clinical status, demographic and socioeconomic characteristics, health behaviors, health service use patterns, local health care supply, and other contextual effects. By testing for group differences with such a comprehensive model, we expected to identify previously unexamined variables that could be readily modified and targeted toward large population groups in such a manner to positively impact their ED use patterns.

In particular, we hypothesized that the one group of older adults, consisting of those who persistently present with clinically severe conditions that require urgent staff attention, is more likely to have higher levels of clinical morbidityand a history of poor health and disability [7-9]. Additionally, we hypothesized that a second group, consisting of those who persistently present with clinically non-severe conditions that do not require immediate care, is more likely to live in areas with poor access to primary care and have lower amounts of service continuity [10-12].

## Methods

### Design

We conducted a secondary analysis of the prospective cohort study known as the Survey on Assets and Health Dynamics among the Oldest Old (AHEAD) [20,21]. AHEAD is a national, omnibus health and retirement longitudinal data source of Medicare-eligible older adults administered by the Survey Research Center at the University of Michigan. AHEAD is a prospective cohort study in which subject interviews have been conducted about every two years since 1993, and the survey questions field a wide array of information including demographics, cognitive performance, physical and functional health, Medicaid eligibility, family structure, care-giving, and out-of-pocket costs for health and social services. Human subjects approval for our study was provided by the AHEAD Restricted Data Use Committee (# 2003-006), the University of Iowa IRB (# 2003-03008), and the Centers for Medicare and Medicaid Services (Data Use Agreement # 14807).

### Sample

Two sampling frames were used in creating AHEAD-a 1992 multi-stage household screening process and a supplemental sample of persons ≥ 80 years old from the Medicare Master Enrollment File. Baseline interviews were conducted in 1993 with 7,447 participants ≥ 70 years old (response rate = 80.4%). We created the analytic sample for this study by linking baseline AHEAD interviews to Medicare inpatient, outpatient, and carrier claims for calendar years 1991-2007. Among the 7,447 older adults who completed the baseline AHEAD interviews in 1993-1994, we excluded 802 because their AHEAD data could not be linked to their Medicare claims. We excluded another 604 participants who were enrolled in managed Medicare at baseline because these plans do not provide comparable claims data. We excluded 531 AHEAD participants who required proxy respondents at the baseline interview and who did not complete cognitive and psychosocial protocols that measured risk factors included in our analysis. The number of AHEAD participants with linked Medicare claims data and included in this analysis totaled 5,510 men and women (74.0% of the original AHEAD sample).

In our previous work concerning heart attack risk and hip fractures, we applied a propensity score analysis to identify potential differences between those individuals who were not included in the analytic sample with those who were selected into the study. Using baseline characteristics available for both groups, we determined that persons not included in the analysis were similar to those that were. Our sample exclusion criteria did not correspond with a selection bias that meaningfully altered the results [22-24].

### Data Collection and Processing

Two CMS Medicare standard analytic files contain data on the provision of care in the ED--outpatient claims files and carrier claims files [25]. ED services provided by physicians employed as hospital staff are submitted by the hospital as outpatient claims, and these claims reflect both professional (i.e., physician effort) and technical (i.e., lab testing) components of ED care. Physicians who are either self-employed or part of a larger, hospital affiliated physician group submit their ED claims to a designated Medicare carrier. In both cases claims submitted for procedures completed during an ED visit are identified with Current Procedural Terminology (CPT) evaluation and management codes 99281-99285. (CPTs refer to the numeric coding system developed by the American Medical Association and used by healthcare providers to document clinical tasks performed during a particular procedure.) This approach to identifying ED claims has previously been used by the Institute of Medicine, and it accounts for over 80% of all Medicare ED expenditures [13].

In our previous study, we delineated a process for bundling these individual Medicare claims into a single episode of ED care consistent with Medicare guidelines [3]. In particular, we bundled outpatient claims for which the "from" and "through" dates overlapped or were within 3 days, consistent with Medicare policy requiring outpatient claims files to be bundled if they occur within 72 hours. For the carrier files, we bundled claims with overlapping dates or those that were within 1 day of each other. This was necessary because Medicare claims have date but not time stamps, and therefore it is possible for a late-night ED encounter to carry over into the next calendar day. We then bundled the outpatient and carrier claims with overlapping dates and defined them as belonging to the same ED episode. We recognized that bundling claims over a consecutive three-day period may underestimate the actual number of episodes given that some individuals may enter and complete an ED episode on one day and then return to the ED on the next day. Therefore, we identified the number of episodes in which all claims were filed in a one day period from those in which claims spanned a two or three day period. This approach represented a significant methodological advancement because we eliminated the over-counting bias that otherwise occurs when ED use is constructed as a discrete visit measured by an individual claim. We used this same bundling approach to define the ED episodes in this study, extending our observation period through 2007.

We then measured the clinical severity of each ED episode using an approach created by Billings et al. [26], then refined by Wharam and his colleagues [27], and recently validated by Ballard et al. [28]. Originally, Billings et al. created an algorithm (i.e., the NYU algorithm) to classify the severity of ED care by using the ICD9-CM diagnostic codes as identified in the ED. Using the diagnostic information, Billings and his colleagues calculated the probability that an ED claim fell into one of four categories: 1) non-emergent (NE); 2) emergent, primary care treatable (EPCT); 3) ED care needed, preventable/avoidable (EDCNPA); and 4) ED care needed, not avoidable (EDCNNPA; http://wagner.nyu.edu/chpsr/index.html). Since administrative records do not contain adequate information to make absolute determinations as to the appropriate category, the original NYU algorithm assigns probabilities that a visit falls into each of the four above categories, yielding four probability estimates.

NE cases are those in which the patient's initial complaint, presenting symptoms, vital signs, medical history, and age indicated that immediate medical care was not required within 12 hours. The EPCT cases are those in which emergent care was required within 12 hours, though the presenting problem did not require continuous observation and no procedures were performed or resources used (i.e., a CT scan or lab work) that were not available in a primary care setting. The EDCNPA cases indicate that emergency department care was required, but the emergent nature of the condition was potentially preventable/avoidable if timely and effective ambulatory care had been received. Finally, EDCNNPA cases are those in which emergency department care was required and ambulatory care treatment could not have prevented the condition. Wharam et al. simplified Billings et al.'s approach by generating a single measure of ED severity based on the summation of probabilities. Their modified-NYU algorithm, which we used in this study, defined a severe visit (modified-NYU rating = 3) as one in which the probability that the ED was needed was ≥ 75% (e.g, EDCNNPA + EDCNPA ≥ 0.75). ED episodes were defined as non-severe (modified-NYU rating = 1) if the probability that ED care was needed was ≤ 25% (i.e. EDCNNPA + EDCNPA ≤ 0.25). ED episodes that did not meet either criteria were defined as indeterminate severity (modified-NYU rating = 2).

We identified 41,739 ED claims among our sample. However, 14,116 (33.8%) of these included primary diagnoses of trauma (n = 8,652), alcohol (n = 21), drug-related (n = 6), psychiatric (n = 560), and 4,877 other diagnoses and were not included in our analysis because Billings et al. did not classify the severity of these types of visits when originally developing their algorithm. This left 27,623 ED claims that were bundled into 20,169 episodes of care.

### Measurement

We measured individual ED use patterns by counting the total number of ED episodes during the observation period and determining if any particular type (severe, non-severe, indeterminate) constituted 50% or more of an individual's total number of episodes. If an individual presented to the ED three times over a six-year observation period and two episodes were rated as severe, then she was grouped with those consistently presenting to the ED with clinically severe episodes. If another individual presented six times in 10 years and three of these episodes were defined as non-severe, two were indeterminate and one was severe, then she was grouped with those who typically present to the ED with non-severe conditions. Individuals who presented with indeterminate levels of severity at least 50% of the time were placed into a third group. Other individuals who did not use the ED or did not display a definitive pattern (had less than 50% of any particular type of episode) during the observation period were not included in the analysis.

### Analysis

We expanded Andersen's model of health service use [19], and considered clinical, demographic and socioeconomic, health behaviors, health status, service use, local health market, and other contextual factors when testing for differences between the two large groups of older Medicare beneficiaries who presented with the most clearly defined and contrasting patterns of ED episodes -- those displaying a persistent pattern of severe ED episodes versus those with a persistent non-severe pattern. In an effort to further isolate the unique drivers of ED use, we contrasted these two groups against the third group of older adults with persistent indeterminate severity ED patterns.

We chose age, race, sex, marital status, and living alone as important demographic factors to include in the statistical models. Socioeconomic characteristics included education, income, and the number of health insurance policies. Health behaviors were represented by body mass index, smoking status, and alcohol use. Functional status assessment included a self-reported health question, median splits on activities of daily living (ADLs) and instrumental ADLs (IADLs), and an 8-item version of the Center for Epidemiologic Studies Depression rating scale (CESD-8) [29]. Cognitive assessment was evaluated using the Telephone Interview to Assess Cognitive Status (TICS) [30]. Disease history was tapped by baseline self-reports of having been told by a physician that one has arthritis, cancer, diabetes, heart attack, heart disease, hypertension, lung disease, or a psychiatric condition. Geographic factors included population density, Census region, the number of hospital beds and physicians per 1,000 persons in the county of residence (from the Area Resource File based on baseline place of residence), distance to the nearest ED, and perceived neighborhood safety. Health services use included the number of physician visits and hospital episodes in the year prior to baseline, and whether the individual maintained continuity of care with a medical provider post-baseline (from claims files).

We created an additional measure which captured the change in an individual's self-rated health from the baseline interview to the interview that preceded the first ED episode. We categorized these individual changes in self-rated health into four groups: those individuals with no change in self-rated health from baseline to the interview prior to the first ED episode; those with decline in self-reported health; those with improved self-reported health; and those in which only one assessment of self-rated health was provided at baseline.

We used multivariable logistic regression to test for differences between the persistent severe and persistent non-severe ED groups, and multinomial logistic regression to contrast these two groups against the third group with persistent indeterminate ED patterns. Our analysis employed three strategies in developing the final statistical models (i.e., forward stepwise selection, forced entry of all potential risk factors and backward elimination). By only selecting variables statistically significant in one or more of these approaches into our final model, we reduced the chance of identifying spurious associations [31]. Model fit was determined by Hosmer-Lemeshow statistics for homoscedasticity [32] and the C-statistic to reflect overall fit (area under the curve) [33]. Finally, to test for differences between individuals with different ED use volumes, we stratified on three or more ED episodes during the observation periodversus less than three and estimated the logistic regressions across and within the severe and non-severe groups.

## Results

Three-fourths (4,271) of the 5,510 AHEAD participants had at least 1 ED episode during the observation period with a mean of 4.7 episodes per person. The volume of ED visits rose slightly over time from 1,451 episodes per 1,000 participants in 1992 to 1,667 episodes per 1000 participants in 2007. This reflects the aging of the AHEAD cohort, inasmuch as the mean age of those having ED episodes increased steadily from 78 to 90 years old during this period.

**Figure 1:**
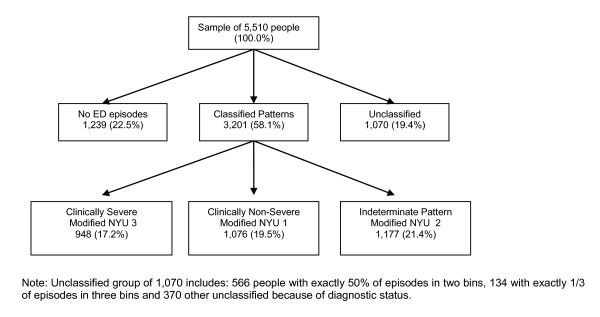
**Patterns of individual ED episodes by group**.

Among the 4,271 who used the ED during the observation period and had a persistent pattern of use, 948 individuals (17.2% of the entire study sample of 5,510) presented a pattern in which their ED episodes were defined as highly severe at least 50% of the time, 1,076 individuals (19.5% of the sample) presented a pattern in which their ED episodes were defined as non-severe at least 50% of the time, and 1,177 (21.4%) presented a pattern in which their episodes were defined as indeterminate at least 50% of the time. Another 1,070 individuals (19.4%) did not display a definitive pattern of ED use and were not classified into any of these three groups. The groups are shown in Figure 1. We also determined that 519 (54.7%) of those with a severe pattern presented to the ED three or more times during the observation period and 661 (61.4%) of those with non-severe patterns presented three or more times.

The results of the logistic regression are shown in Table 1. In the second column, the odds ratios and confidence intervals are presented for individuals with a persistently severe pattern of ED episodes compared to those with persistently non-severe patterns. Compared to persons with persistently non-severe patterns, the group with persistently severe pattern of ED episodes was more likely to be older (AOR 1.52; CI 1.23-1.87),  be men (AOR 1.28; CI 1.02-1.60), be current smokers (AOR 1.60; CI 1.12-2.28), have experienced diabetes (AOR 1.80; CI 1.32-2.44), heart disease (AOR 1.70; CI 1.33-2.17), and hypertension (AOR 1.32; CI 1.09-1.61), and have four or more morbid conditions (AOR 1.48; CI 1.00-2.20). The Hosmer-Lemeshow statistic was 6.0 (p = 0.64), and the C-statistic was 0.68, indicating good model fit.

**Table 1:** Logistic regression predicting ED episode patterns

	All Episodes		< 3 Episodes		≥ 3 Episodes	
	**AOR**	**95% CI**	**AOR 95% CI**		**AOR**	**95% CI**

Age Above Median	1.520	(1.234, 1.873)	1.267	(0.901, 1.781)	1.728	(1.298, 2.299)
African American	0.784	(0.561, 1.096)	0.588	(0.340, 1.018)	1.020	(0.648,1.604)
Hispanic	0.895	(0.532, 1.508)	0.833	(0.298,2.326)	1.248	(0.662, 2.335)
White (ref)						
Men	1.280	(1.022, 1.604)	1.788	(1.248, 2.562)	1.006	(0.736, 1.375)
Lives Alone	1.195	(0.966, 1.478)	1.393	(0.990, 1.962)	1.119	(0.835, 1.498)
# of Insur Health Policies	0.947	(0.808, 1.109)	0.610	(0.470, 0.792)	1.323	(1.059, 1.653)
Body-Mass Index: Obese	1.013	(0.991, 1.035)	1.002	(0.965, 1.041)	1.022	(0.993, 1.051)
Never Smoked (ref)						
Former Smoker	1.175	(0.947, 1.459)	1.149	(0.812, 1.626)	1.236	(0.920, 1.662)
Current Smoker	1.601	(1.124, 2.280)	1.998	(1.171, 3.407)	1.447	(0.871, 2.405)
No Drinks (ref)						
Between 1&2 Drinks	0.692	(0.480, 0.998)	0.759	(0.436, 1.324)	0.651	(0.386, 1.097)
More than 3 Drinks	0.903	(0.446, 1.828)	0.744	(0.287, 1.933)	1.352	(0.411, 4.440)
Self-Reported Health (VG)						
Fair Self-Reported Health	1.052	(0.820, 1.349)	1.101	(0.737, 1.644)	0.955	(0.680, 1.343)
Poor Self-Reported Health	1.207	(0.846, 1.722)	0.902	(0.471, 1.729)	1.363	(0.864, 2.150)
IADL 1 or More	1.021	(0.785, 1.327)	1.401	(0.915, 2.145)	0.820	(0.570, 1.180)
Arthritis	0.605	(0.479, 0.764)	0.856	(0.581, 1.262)	0.454	(0.331, 0.623)
Diabetes	1.795	(1.323, 2.436)	3.559	(1.986, 6.378)	1.250	(0.847, 1.846)
Heart Disease	1.699	(1.334, 2.165)	1.550	(1.051, 2.285)	1.790	(1.293, 2.478)
Hypertension	1.323	(1.085, 1.612)	1.408	(1.086, 2.068)	1.222	(0.934, 1.598)
Lung Disease	0.727	(0.504, 1.409)	0.582	(0.316, 1.074)	0.900	(0.557, 1.455)
Psych. Problems	0.997	(0.685, 1.451)	1.348	(0.682, 2.663)	0.803	(0.495. 1,300)
Stroke	0.787	(0.570, 1.084)	0.970	(0.559, 1.681)	0.741	(0.487, 1.127)
4 or More Morbid Conditions	1.484	(1.001, 2.199)	1.014	(0.479, 2.146)	1.896	(1.155, 3.113)
Hospitalized in last 12 mo.	1.260	(0.995, 1.596)	1.281	(0.848, 1.934)	1.213	(0.893, 1.648)
Visits to MDs in 2 yrs	1.001	(0.983, 1.020)	1.016	(0.977, 1.056)	0.990	(0.968, 1.013)
Upper 20% beds per 1,000	0.780	(0.598, 1.018)	0.855	(0.558, 1.310)	0.646	(0.448, 0.930)
Upper 20% MDs per 1,000	1.096	(0.841, 1.428)	1.486	(0.973, 2.270)	0.853	(0.595, 1.223)
Distance to ED	0.992	(0.981, 1.003)	0.985	(0.966, 1.004)	0.993	(0.979, 1.008)
Population > 1 mln	0.987	(0.793, 1.229)	0.826	(0.581, 1.172)	1.091	(0.810, 1.470)
Population < 20,000	0.863	(0.650, 1.146)	0.784	(0.49, 1.257)	0.908	(0.621, 1.328)
Years from first to last ED	0.947	(0.922, 0.972)	0.988	(0.892, 1.093)	0.916	(0.882, 0.951)

Note: The model also included income quintiles, the change in self-rated health measure,

self-rated memory, self-reported neighborhood safety and measure of the continuity of care, none of which were statistically significant, and are not shown here due to space constraints. Their inclusion or exclusion did not alter the parameters shown in the table

The third and fourth columns of the table contain results comparing the severe and non-severe groups based on the frequency of ED episodes. Among individuals who presented less than three times, a persistent pattern of severe ED episodes was more likely for men (AOR 1.79; CI 1.25-2.56), current smokers (AOR 2.00; CI 1.17-3.41), and those with diabetes (AOR 3.56; CI 1.99-6.38), heart disease (AOR 1.55; CI 1.05-2.29), and hypertension (AOR 1.50; CI 1.09-2.07), and less likely for those who had health insurance policies in addition to their basic Medicare coverage (AOR 0.61; CI 0.47-0.79). The Hosmer-Lemeshow statistic was 10.3 (p = 0.24), and the C-statistic was 0.69, indicating good model fit. The third column of the table contains the results pertaining to individuals who presented to the ED three or more times. Among individuals with a higher volume of ED episodes, a persistent pattern of severe ED visits was more likely to occur for older adults (AOR 1.73; CI 1.30-2.30), for those who experienced four or more morbid conditions (AOR 1.90; CI 1.16-3.11), had heart disease (AOR 1.79; CI 1.29-2.48), and carried health insurance policies in addition to their Medicare coverage (AOR 1.32; CI 1.06-1.65), while being less likely for those in an area with a high concentration of hospital beds (AOR 0.65; CI 0.45-0.93). The Hosmer-Lemeshow statistic was 9.73 (p = 0.28) and the C-statistic was 0.70, indicating good model fit.

We also tested for within group differences in terms of frequency (not shown in Table 1). Persons who persistently presented with severe conditions but had fewer than three ED episodes during the observation period were less likely to be older (AOR 0.56; CI 0.36-0.88), to be in poor health (AOR 0.44; CI 0.22- 0.88) and less likely to experience hypertension (AOR 0.59; CI 0.38-0.92) or lung disease (AOR 0.48; CI 0.23-0.99) than those who had three or more ED episodes. Persons who persistently presented with non-severe conditions but had fewer than three ED episodes during the observation period were less likely to have difficulty with activities of daily living (AOR 0.51; CI 0.30-0.87) than those who had three or ED episodes during the observation period.

The results of the multinomial logistic regression are shown in Table 2. Compared to persons with persistent indeterminate patterns, individuals with a persistent severe pattern of ED episodes were more likely to be older (AOR 1.36; CI 1.13-1.65), more likely to be obese (AOR 1.36; CI 1.03-1.82), and to experience heart disease (AOR 1.49; CI 1.20-1.87) and hypertension (AOR 1.36; CI 1.13-1.64). These individuals also were more likely to have been hospitalized (AOR 1.29; CI 1.03-1.60) and live in rural areas (1.33 CI 1.00-1.77). Individuals with a persistent non-severe pattern of ED episodes were less likely to be men (AOR 0.79; CI 0.64-0.96) and current smokers (AOR 0.63; CI 0.46-0.87) than persons with persistent indeterminate patterns; they also were less likely to have diabetes (AOR 0.67; CI 0.50-0.88) or lung disease (AOR 0.58; CI 0.43-0.79). These individuals were more likely to moderately consume alcohol (AOR 1.42; CI 1.03-1.94) and experience arthritis (AOR 1.28; CI 1.04-1.57) or a stroke (AOR 1.70; CI 1.24-2.32). They also were more likely to live in rural areas (AOR 1.48 CI 1.14-1.92).

**Table 2:** Multinomial logistic regression predicting ED episode patterns

	Severe Group (> 50% = NYU 3)		Non-Severe Group (< 50% = NYU 1)	
	**AOR**	**95% CI**	**AOR**	**95% CI**

Age Above Median	1.363	(1.125, 1.653)	0.934	(0.777, 1.122)
African American	0.812	(0.591, 1.116)	1.127	(0.842, 1.508)
Hispanic	0.847	(0.518, 1.386)	1.066	(0.679, 1.674)
White (ref)				
Men	1.026	(0.832, 1.265)	0.788	(0,644, 0.964)
Lives Alone	1.065	(0.877, 1.294)	0.927	(0.770, 1.116)
# of Insur Health Policies	0.968	(0.833, 1.125)	0.991	(0.859, 1.144)
Body Mass Index: Obese	1.364	(1.025, 1.815)	1.199	(0.916, 1.571)
Never Smoked (ref)				
Former Smoker	0.968	(0.786, 1.191)	0.823	(0.676, 1.002)
Current Smoker	0.908	(0.660, 1.248)	0.633	(0.462, 0.866)
No Drinks (ref)				
Between 1&2 Drinks	1.027	(0.720, 1.465)	1.417	(1.032, 1.945)
More than 3 Drinks	1.108	(0.568, 2.163)	1.197	(0.637, 2.250)
Self-Reported Health (VG)				
Fair Self-Reported Health	1.0465	(0.834, 1.312)	1.005	(0.808, 1.248)
Poor Self-Reported Health	1.230	(0.894, 1.693)	1.061	(0.772, 1.459)
IADL 1 or More	0.902	(0.705, 1.154)	0.954	(0.751, 1.213)
Arthritis	0.785	(0.626, 0.984)	1.281	(1.044, 1.572)
Diabetes	1.095	(0.839, 1.428)	0.665	(0.502, 0.882)
Heart Disease	1.494	(1.195, 1.867)	0.974	(0.780, 1.215)
Hypertension	1.361	(1,128, 1.643)	1.006	(0.842, 1.203)
Lung Disease	0.392	(0.285, 0.541)	0.580	(0.432, 0.778)
Psych. Problems	0.873	(0.612, 1.245)	0.951	(0.682, 1.326)
Stroke	1.322	(0.962, 1.817)	1.698	(1.242, 2.321)
4 or More Morbid Conditions	1.152	(0.816, 1.626)	0.758	(0.528, 1.088)
Hospitalized in last 12 mo.	1.285	(1.029, 1.604)	1.003	(0.806, 1.248)
Visits to MDs in 2 yrs	0.995	(0.979, 1.011)	0.999	(0.983, 1.015)
Upper 20% beds per 1,000	0.780	(0.603, 1.008)	1.026	(0.810, 1.300)
Upper 20% MDs per 1,000	1.132	(0.881, 1.454)	1.009	(0.794, 1.283)
Distance to ED	0.993	(0.983, 1.004)	0.999	(0.989, 1.009)
Population > 1 mln	1.027	(0.836, 1.262)	1.039	(0.853, 1.265)
Population < 20,000	1.331	(1.004, 1.765)	1.479	(1.139, 1.920)
Years from first to last ED	0.929	(0.907, 0.951)	0.998	(0.978, 1.019)

## Discussion

We observed a pluralistic pattern of ED use among a nationally representative sample of older adults. Nearly 1 out of every 5 (17.2%) older adults persistently presented to the ED with clinically severe conditions; a second group constituting an additional 20% of persons over 65 persistently presented with non-severe conditions; and a third group representing slightly more than 21% of older adults persistently presented with a pattern of indeterminate episodes. These groups differed from each other in significant ways. Individuals with a persistent pattern of presenting to the ED with severe episodes were more likely to be older, male, and have chronic disease conditions, such as diabetes, heart disease, and hypertension, and greater levels of morbidity than those with persistent patterns of non-severe and indeterminate ED episodes. These findings provide more evidence for empirically well-established associations among age, health and service use [34].

In considering these results further, we observed that cardiac dysrhythmias and heart failure accounted for more than 30% of the severe episodes, suggesting that as individuals with heart disease aged, they acquired conditions which contributed toward persistently presenting to the ED with clinically severe needs. Assuming that the ED remains a primary point of service contact for this large group of older individuals with severe clinical problems, the demand placed on staff and resources will become substantial as the aging population grows in the coming decade [4]. As such, ED providers should consider developing geriatric protocols and increasing the supply of staff trained in geriatric assessment and care management [2,35]. Alternatively, efforts could be directed toward developing population management programs that reduce ED use among those with diabetes, heart disease, and other co-morbidities that are associated with persistently severe ED patterns. For example, by enrolling persons with diabetes into patient education programs or enlisting people with cardiovascular disease into chronic disease management programs, the patterns of older individuals who persistently use the ED for severe conditions may be beneficially altered as they have been with other populations [36-38].

We hypothesized that individuals who persistently use the ED for less severe episodes would differ in terms of their access to services and continuity of care, expecting our findings would support efforts that focus on readily modifiable variables as a way to address the excess demand created by this large group. Although we found that both the persistently severe and persistently non-severe groups were more likely to live in rural areas than persons with persistently indeterminate patterns of ED use, we found no other significant differences in terms of local service supply or continuity of care.

We also hypothesized group differences might be related to potentially modifiable, previously unexamined health behaviors such as obesity or smoking. Our results, however, did not provide a clear picture. Persons with persistently severe patterns of ED use were more likely to be current smokers than those with persistently non-severe patterns, but they were no different than those with persistently indeterminate patterns. Alternatively, persons with persistently severe patterns were more likely to be obese compared to persons with persistently indeterminate episodes, but they were no more likely to be obese than those with persistently non-severe patterns.

Still our (lack of) findings provide some support that older individuals who persistently bring less severe conditions to the ED may be electing to bypass readily available community-based alternatives. In particular, given that we found no differences between the groups in terms of education, income and supplemental insurance coverage, it is plausible to contend that the nearly universal coverage afforded by Medicare has mitigated any sensitivity older adults may have to deductible and co-payment obligations, and thus, perhaps they view their use of the ED comparably to their use of primary care [28,37]. Indeed, others have contended that the ED has become a substitute for primary care because individuals have resolved that the ED is comparable if not superior to primary care, offering immediate access, a full range of diagnostic and treatment services, and a more definitive resolution to their presenting complaint [39].

If this large and growing population of older adults is in fact insensitive to using the ED relative to primary care, then their demand for the ED will continue to increase. Perhaps future research efforts could examine how altering deductible and co-payment obligations might affect service use patterns so that those who persistently present with non-severe conditions become more sensitive about using the ED when community-based alternatives are available [12,40].

Finally, in comparing across and within the persistently severe and persistently non-severe groups based on the frequency of ED use, our results indicated that the differences were more definitive among those with fewer ED episodes. As a person aged and the frequency of ED episodes increased, the group differences became less pronounced. These particular findings substantiate the difficulty in trying to develop a management approach that targets individual visits at any given point in time.

This study is not without limitations. First, no data were available on the EDs to which the individuals presented, and we were unable to reduce the heterogeneity of these EDs in terms of capacity, staffing, and procedural policies. Second, although we noted there were other older ED users in the AHEAD sample, our analysis was limited in that we did not include ED use for trauma, drug, and alcohol situations and did not include these persons in the group comparisons.

Nonetheless our work further illuminates the intersection between older adults and their use of the ED. In observing ED episodes over an extended observation period and testing an expanded model, we affirmed previous findings about the relationship between age, health status and persistent use of the ED for severe conditions. Future research might examine how targeted efforts to manage population groups that persistently present with severe conditions might alter ED use. We also found that while older adults who persistently present to the ED with non-severe conditions may not experience problems with access and continuity or clearly differ in terms of modifiable health behaviors, they may be price insensitive and consider the ED as a substitute for primary care. Perhaps future research could further examine insurance and claims data and test if variations in coverage corresponded with differing patterns of ED use for other groups (i.e., Medicaid eligibles) who persistently present with non-severe conditions.

## Conclusion

We distinguished three readily identifiable groups of older adults who figure prominently in the use of the ED. As more Americans reach and surpass their 65^th ^birthdays, one group (those persistently presenting with clinically severe ED episodes) will increasingly affect the general capacity of the ED to provide high quality, cost effective health care as these individuals present with more severe episodes requiring urgent staff attention and greater resource allocation. In contrast, a second group (those who persistently present with non-severe conditions) will assume an increasingly larger share of those who use of the ED as a possible substitute for other community-based services. Future research should consider how managing chronic health conditions or adjusting co-payment obligations might impact the use of the ED among the growing older adult population.

## Competing interests

The authors declare that they have no competing interests.

## Authors' contributions

FDW conceived of the study, wrote the grant application, designed the analyses, interpreted the results, and revised the manuscript. RLO, GER, and RBW participated in the conceptualization of the grant application and the overall study design, and provided their expertise throughout the study. MO constructed episodes of ED use, constructed measures of severity, completed statistical analyses, and prepared the manuscript. SEB & MO assisted with analyses. BK contributed to study design, assisted with statistical analyses, interpreted the results, and prepared the manuscript. MO assisted with all data linkage, merging, and recoding. All authors participated in numerous meetings to outline, read, critique, revise, re-read, and grant approval the final manuscript. FDW, BK, and MO had full access to all of the data in the study and take responsibility for the integrity of the data and the accuracy of the data analysis. All authors read and approved the final manuscript.

## Pre-publication history

The pre-publication history for this paper can be accessed here:

http://www.biomedcentral.com/1471-2318/11/65/prepub

## Acknowldegements

Supported by NIH grants R01 AG-022913, R21 AG-030333, and R21 AG-031307 to Dr. Wolinsky. Dr. Jones is the Senior Statistician and Dr. Hockenberry is an affiliated investigator at CADRE. CADRE is funded through the Department of Veterans Affairs, Veterans Health Administration, Health Services Research and Development Service (HFP 04-149). Dr. Obrizan would like to thank the Victor Pinchuk foundation, EERC and Stockholm Institute of Transition Economics (SITE) for their support. The opinions expressed here are those of the authors and do not necessarily reflect those of any of the funding, academic or governmental institutions involved.
